# N-acetyltransferase 2 genetic polymorphisms and anti-tuberculosis-drug-induced liver injury: a correlation study

**DOI:** 10.3389/fphar.2023.1171353

**Published:** 2023-08-31

**Authors:** Fang Cheng, Xian-Gao Jiang, Shi-Lin Zheng, Te Wu, Qiang Zhang, Xin-Chun Ye, Saiduo Liu, Ji-Chan Shi

**Affiliations:** Department of Infectious Disease of Wenzhou Central Hospital, Wenzhou Central Hospital, The Dingli Clinical College of Wenzhou Medical University, Wenzhou, China

**Keywords:** NAT2, gene polymorphisms, anti-tuberculosis-drug-induced liver injury, tuberculosis, correlation study

## Abstract

**Background:** Considering the genetic characteristics of people with anti-tuberculosis (TB)-drug-induced liver injury (ATDILI), genetic factors and their consequences for treatment need to be studied.

**Objective:** The correlation between N-acetyltransferase 2 (NAT2) genetic polymorphisms and ATDILI was analysed.

**Methods:** In this study, the liver and coagulation functions of 120 patients with TB were monitored dynamically for at least 3 months. The genetic polymorphisms of patients were detected by pyrosequencing, and the acetylation types of liver damage and the distribution of NAT2 genetic polymorphisms were compared and analysed.

**Results:** The results showed that there were significant differences in the distribution of alleles and acetylation types among different groups (*p* < 0.05). In patients with grade 4 liver injury (liver failure), any two alleles were included, i.e., *6 and *7. Specifically, patients with fast acetylation genotypes accounted for 42.4% (14/33), those with intermediate acetylated genotypes accounted for 55.2% (32/58), and patients with slow acetylation genotypes accounted for 65.5% (19/29).

**Conclusion:** Patients with slow acetylation genotypes had higher rates of liver failure and liver injury than those with intermediate and fast acetylation genotypes, and patients with slow acetylation genotypes containing any two alleles (*6 and *7) had a higher rate of liver failure than those with other alleles. In summary, the time of liver injury in patients with slow acetylation genotypes was earlier than the total average time, and the time of liver function recovery in patients with fast acetylation genotypes was shorter than the total average time.

## Highlights

1. N-acetyltransferase 2 (NAT2) genetic polymorphisms were related to anti-tuberculosis (TB) -drug-induced liver injury in Chinese patients with TB.

2. Patients with TB with slow acetylation genotypes had higher rates of liver injury.

3. Patients with TB with slow acetylation genotypes had higher rates of liver failure.

4. Patients with TB and alleles *6 and *7 had higher rates of liver failure. fx1fx2

## Introduction

According to the *Global Tuberculosis Report 2022* released by the WHO, an estimated 10.6 million people became ill with tuberculosis (TB) in 2021, compared with 10.1 million in 2020, and 1.6 million people died from TB in 2021, compared with 1.5s million in 2020 (Bagcchi, 2023). Isoniazid (INH), rifampicin (RFP), pyrazinamide (PZA) and ethambutol (EMB) are the first-line medications used in traditional anti-TB therapy, and all are metabolised by the liver, which may lead to the development of anti-TB-drug-induced liver injury (ATDILI). Deaths caused by ATDILI are uncommon but possible (Wang et al., 2016; Jiang et al., 2021; Zhou et al., 2022). The risk of liver damage during treatment can vary significantly between individuals and, accordingly, refers to the issue of individual susceptibility (Kim et al., 2010; Bose et al., 2011; Chen et al., 2015).

The occurrence of ATDILI is related to the production and elimination of toxic substances during drug metabolism in the liver, where INH is the most prominent first-line anti-TB drug that causes drug-related liver injury (Huang, 2014). Isoniazid is metabolised *in vivo* by N-acetyltransferase 2 (NAT2) to produce intermediate products, such as acetyl INH, isonicotinic acid and acetyl hydrazide, which, ultimately, produce non-toxic diacetyl hydrazine. Hydrazine and ketene, which are produced in this metabolic pathway, are hepatotoxic substances that can cause drug-related liver injury (Huang et al., 2002; Saukkonen et al., 2006; Huang, 2007; Tostmann et al., 2008).

NAT2 genetic polymorphisms affect NAT2 activity and can thus lead to the risk of drug-related liver injury in patients with TB (Zhang et al., 2018). Several papers have reported a correlation between NAT2 genetic polymorphisms and ATDILI and posited that the slow acetylation of NAT2 was significantly associated with the risk of ATDILI (Ng et al., 2014; Beijing Chest HospitalCapital Medical University, 2021; Yang et al., 2022). However, studies correlating NAT2 genetic polymorphisms with ATDILI in China are rare. In addition, it has been reported that a personalised clinical drug dosage model can be developed for TB treatment, which is especially important for those areas of South and East Asia with high incidences of ATDILI (Bagcchi, 2023). Therefore, we aimed to investigate the association of NAT2 genetic polymorphisms with ATDILI in Chinese patients with TB.

The distinct *NAT2* genotype can be divided into three types, i.e., fast, intermediate and slow acetylation genotypes. According to the results of genetic tests based on previous research, among four mutant loci of *NAT2* genes (C282T, T341C, G590A and G857A), 282 loci were typically mutated in combination with 590 or 857 to form alleles *6 and *7; 341 loci were mutated to form allele *5; and four loci that were not mutated were wild-type allele *4. According to the four denoted alleles, *NAT2* could be classified into the wild homozygous fast acetyl type, i.e., *4/*4; the wild mutant heterozygous intermediate acetyl type, i.e., *4/*5, *4/*6 and *4/*7 and the mutant homozygous slow acetyl type, i.e., containing any two of alleles *5, *6 or *7 (Dong et al., 2020); thereby, we evaluated the association of NAT2 genetic polymorphisms in three different genotypes with ATDILI in TB based on the above protocols.

Overall, we hypothesised that NAT2 genetic polymorphisms are associated with ATDILI.

## Participants and method

### Subjects

The inclusion criteria were as follows: 1) patients aged 16–85 years; 2) a clear diagnosis of primary TB treatment, including patients with pathological confirmation, pathogenetic confirmation and clinical diagnosis; 3) normal liver function before anti-TB treatment and 4) patients who provided signed informed consent for their voluntary inclusion in the study.

**FIGURE. 1:**
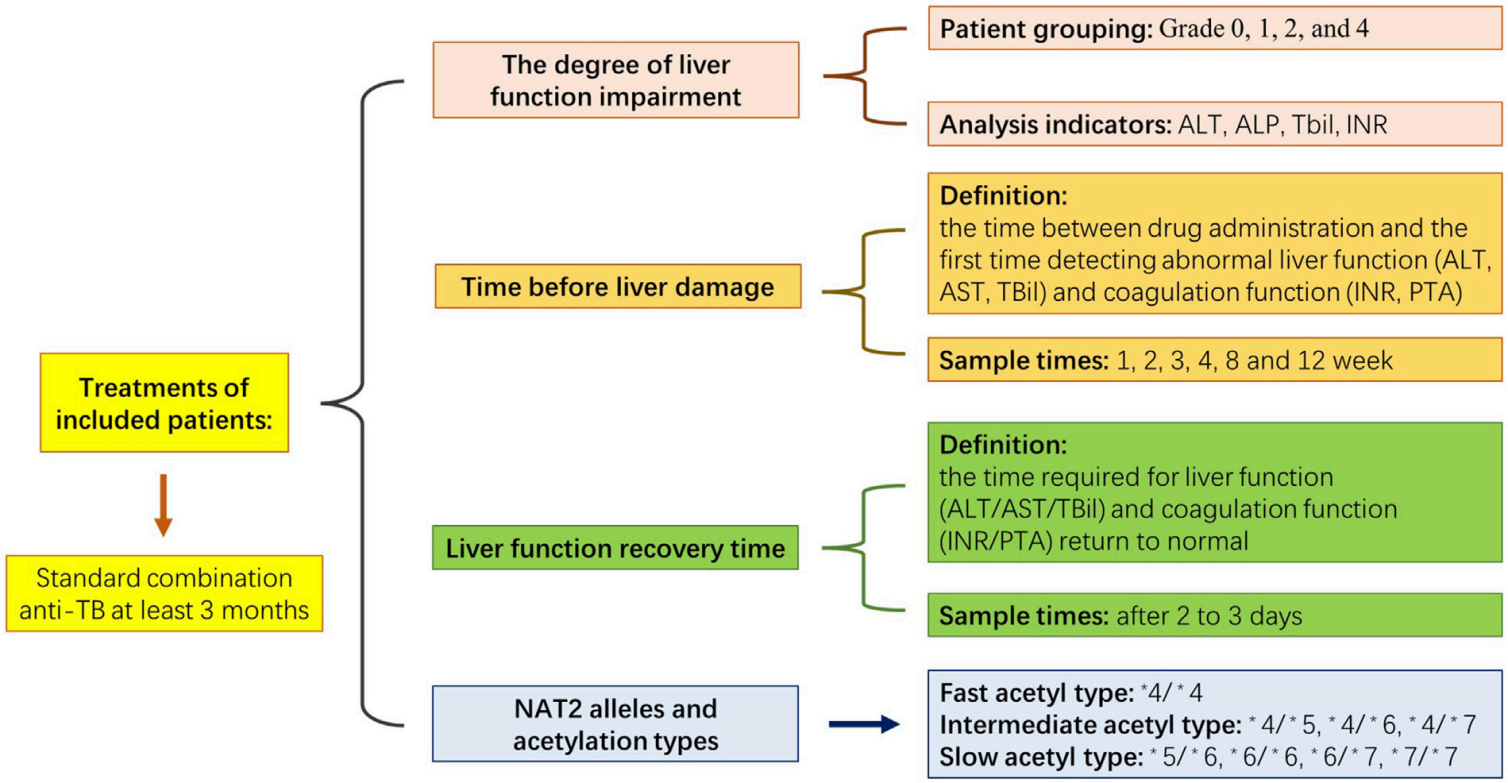
The schedule for the experiment.

The study’s exclusion criteria were as follows: 1) abnormal liver function before anti-TB treatment; 2) patients with other diseases that could cause abnormal liver function, including alcoholic and viral hepatitis, cirrhosis, immune haemolytic disease and congestive heart failure; 3) patients who were taking other drugs that could cause abnormal liver function, including immunosuppressants, anti-tumour drugs, acetaminophen and chlorpromazine; 4) patients who had not completed 3 months of anti-TB treatment for reasons other than liver impairment and 5) patients with abnormal coagulation function before anti-TB treatment. The selection criteria were based on existing research (Dong et al., 2020). The present study was approved by the Ethics Committee of Wenzhou Central Hospital (No. K2020-04-003), and the corresponding documents are listed in Supplementary Table S1. Figure 1 displays the schedule for the experiment.

According to the diagnosis of TB, the presence of TB in 120 patients was confirmed; they included 88 males and 32 females, aged 18–82 years, with a mean age of 55.5 years. The cases included 100 cases of pulmonary TB, six cases of extrapulmonary TB (one case of tuberculous meningitis, three cases of tuberculous pleurisy, one case of lymphatic TB and one case of tuberculous abscess) and 14 cases of pulmonary TB combined with extrapulmonary TB. In addition, there were eight cases of pulmonary TB combined with tuberculous pleurisy, one case of pulmonary TB combined with pelvic TB, two cases of pulmonary TB combined with cervical lymphatic TB, two cases of pulmonary TB combined with laryngeal nodules and one case of pulmonary TB combined with intestinal TB. Among them, 33 patients (all with grade-1 liver injury) developed adaptation to treatment, i.e., mild liver profile elevations that normalised despite treatment continuation. Thirty-two patients (patients with grades 1, 2 and 4 liver injury) changed their anti-TB regimen due to elevated liver indexes.

The general conditions of the study participants are listed in Table 1. The ‘yes’ and ‘no’ values for diabetes mellitus and hypertension in Table 1 represent patients with and without the respective disease. All 120 patients in this study were inpatients. The doctors in charge asked about their medical history in detail and the use of hypotensive and hypoglycaemic drugs. The patients’ blood pressure and blood glucose levels were monitored during hospitalisation.

**TABLE 1:** Comparison of clinical baseline data between the groups with different degrees of liver impairment and the control group (groups without liver impairment).

Variables	Total (n = 120)	0 (n = 55)	1 (n = 55)	2 (n = 7)	4 (n = 3)	F/Z/χ^2^	*P*
sex						Fisher	0.379
man	88 (73.3)	42 (76.4)	39 (70.9)	6 (85.7)	1 (33.3)		
woman	32 (26.7)	13 (23.6)	16 (29.1)	1 (14.3)	2 (66.7)		
age	55.5 (36, 66)	56 (41.5, 65.5)	53 (36, 67)	58 (53.5, 70.5)	25 (21, 45.5)	2.366	0.500
diabetes mellitus						Fisher	0.615
deny	95 (79.2)	43 (78.2)	42 (76.4)	7 (100)	3 (100)		
yes	25 (20.8)	12 (21.8)	13 (23.6)	0 (0)	0 (0)		
hypertension						Fisher	0.782
deny	95 (79.2)	45 (81.8)	42 (76.4)	6 (85.7)	2 (66.7)		
yes	25 (20.8)	10 (18.2)	13 (23.6)	1 (14.3)	1 (33.3)		
ALT (U/L)	14 (9, 22)	11 (7.5, 15.5)	17 (11, 27)	14 (9, 32)	8 (6.5, 17.5)	12.727	0.005
AST (U/L)	19 (14.5, 26)	16 (13.5, 22)	22 (17, 27.75)	26 (16.5, 26.5)	20 (17, 29.5)	11.084	0.011
Tbil (μmol/L)	10.4 (7.3, 14.95)	9.9 (6.25, 13.7)	10.35 (7.45, 14.12)	13.1 (11.75, 22.7)	15.1 (14.15, 16.55)	8.294	0.040
INR	1.04 (0.97, 1.07)	1.04 (0.98, 1.08)	1.03 (0.97, 1.06)	1.02 (0.96, 1.11)	1.04 (1.04, 1.06)	0.810	0.847
PTA (%)	104.2 ± 12.5	102.9 ± 11.8	104.7 ± 12.2	106 ± 22.2	106.7 ± 15.3	0.199	0.896

Note: ALT, alanine aminotransferase; AST, glutamic oxalacetic transaminase; TBil, total bilirubin; INR, international normalised ratio; PTA, prothrombin time activity. Normal range: ALT (7–40 U/L); AST (13–35 U/L); Tbil (0–21 μmol/L); INR (0.8–1.1); PTA (60%–160%).

As noted in the above research methods (Dong et al., 2020), a standard combination anti-TB regimen was implemented for at least 3 months, and all patients received first-line anti-TB drugs according to the following regimen: 2HRZE/4∼7HR (i.e., two months of intensification and four to 7 months of consolidation), with consistent drug doses as follows: INH 300 mg, once daily; RFP 450 mg, once daily; EMB 750 mg, once daily and PZA 500 mg three times daily. The treatments were adjusted accordingly if any patient developed definitive ATDILI.

### N-acetyltransferase genetic polymorphism detection steps and determination methods

Specific whole-blood deoxyribonucleic acid (DNA) extraction was conducted using standard procedures described in existing studies (Zhong et al., 2018). The manufacturer’s instructions for using a QIAamp DNA Blood Mini Kit (Cat. No. 51104; Qiagen, Valencia, CA, USA) were followed. A venous blood sample (2 mL) was collected from each patient in the early morning under fasting conditions using an EDTA-K2 anticoagulated blood collection tube, which was immediately mixed and sent for DNA extraction. Finally, the DNA content was assessed using a NanoDrop 2000™ spectrophotometer (ThermoFisher Scientific, Waltham, MA).

Information about the *NAT2* gene and mutant loci was obtained from the PubMed literature database (www.ncbi.nlm.nih.gov), and primers for pyrophosphate sequencing of the corresponding *NAT2* gene at loci 282, 341, 590 and 857 were designed using PyroMark Assay Design (version 2.0) software. The specific design process that was implemented was adopted from an earlier report (Dong et al., 2020). The primers are listed in Supplementary Table S1.

The experiments involving polymerase chain reaction amplification, single-strand DNA template preparation and pyrophosphate sequencing were performed as described previously (Zhong et al., 2018; Renu et al., 2021).

### Observation indicators

Observation indicator tests were conducted following the standard procedure described in existing studies (Zhong et al., 2018). Patients in each group were tested for alanine aminotransferase (ALT), alkaline phosphatase (ALP), total bilirubin (TBil), prothrombin concentration (PT), glutamic oxaloacetic transaminase (AST)and prothrombin activity using fully automated biochemical analysers (iChem-520; KuBeier, Shenzhen, China) at weeks 2, 4, 8 and 12 of anti-TB treatment. The international normalised ratio (INR) is a blood coagulation index used to monitor the therapeutic effect in patients taking oral anticoagulant drugs. The calculation method for INR detection divides the patient’s PT value by the mean PT value of the control plasma used in the laboratory to obtain a ratio. Then, the ratio is corrected using the INR formula: INR = (patient ratio) ^ International Sensitivity Index.

Time before liver damage was the time between drug administration and the first time detecting abnormal liver function (ALT/glutamic oxaloacetic transaminase [AST]/TBil) and coagulation function (INR/PTA). Generally, the tests are performed at weeks 1, 2, 3, 4, 8 and 12 of treatment. However, when patients showed symptoms of liver damage or adverse drug reactions, such as nausea, vomiting, rash or other symptoms, we immediately conducted liver function and coagulation tests. Part of the test was early or late due to the rest day involved, and the measurement units were in days. Time of liver function recovery: For patients with liver injury, we strengthened the monitoring of liver function and coagulation function and reviewed them after two to 3 days. The calculated time was the time required for the patient’s liver function (ALT/AST/TBil) and coagulation function (INR/PTA) to return to normal, and the measurement units were in days.

### Detecting the degree of liver function injury impairment

The reference standard for the degree of liver function impairment (*Guidelines for the Diagnosis and Treatment of Anti-Tuberculous Drug-Induced Liver Injury, 2019*) (The Society of TuberculosisChinese Medical Association, 2019) was adopted to detect the degree of liver function injury impairment. The levels of ALT, ALP, TBil and INR in the patients were detected using fully automated biochemical analysers (iChem-520; KuBeier, Shenzhen, China) to review the degree of liver injury as follows:

**Grade 1** (mild liver injury): the recoverable elevation of serum ALT and/or serum ALP; TBil <2.5 times the upper limit of normal (ULN) (42.8 μmol/L); INR <1.5 G.

**Grade 2** (moderate liver injury): elevated serum ALT and/or ALP; TBil ≥2.5 times ULN or INR ≥1.5 despite the absence of elevated TBil.

**Grade 3** (severe liver injury): elevated serum ALT and/or ALP; TBil ≥5 times ULN (50 mg/L or 85.5 μmol/L), with or without INR ≥1.5.

**Grade 4** (liver failure): elevated serum ALT and/or ALP levels; TBil ≥10 times ULN (171 μmol/L) or daily elevation ≥10 mg/L or 17.17 mol/L; INR ≥2.0 or prothrombin time activity (PTA) < 40%, possibly with ascites, hepatic encephalopathy or other organ failure associated with ATDILI.

**Grade 5** (fatal): death due to ATDILI or the need to undergo a liver transplant to survive.

### Statistical analysis

This study used SPSS (version 24.0, IBM) statistical software to conduct a statistical analysis. Measurement data that obeyed a normal distribution were described as means ± standard deviations, and a one-way analysis of variance was used for comparisons between multiple groups. Measurement data that did not obey a normal distribution were described by medians and interquartile spacing, and a Kruskal–Wallis H rank–sum test was used for comparisons between multiple groups. Paired samples were compared using a Wilcoxon rank–sum test, while count data were described by frequency. The Chi-squared test or Fisher’s exact probability method was used to analyse the distribution differences, and *p* < 0.05 was considered statistically significant. We estimated the sample size for the distribution rate of acetylation types among liver function groups in the Chi-squared test using PASS software (version 15.0) (NCSS, LLC. Kaysville, Utah, USA). A sample size of 35 achieved 90% power to detect an effect size (W) of 0.7072 using a six-degrees-of-freedom Chi-squared test with a significance level (alpha) of 0.05.

## Results

In this study, the authors hypothesised that NAT2 genetic polymorphisms were associated with ATDILI. This section presents a comparison of the basic clinical data of all the participants and excludes other interfering factors. Additionally, the relationship between different alleles and acetylation types and the degree of liver injury in patients is explored. To study the relationship between the degree of liver function and coagulation function, the authors measured the coagulation index of the patients. Finally, the authors investigated the relationship between different acetylation types and the occurrence and recovery time of liver injury in patients.

### Comparison of clinical baseline data

The general conditions of the study participants were analysed, and the results showed that differences in gender, age, diabetes mellitus, hypertension, INR and PTA were not statistically significant between the different groups (*p >* 0.05). Additionally, although there were statistical differences in AST and TBil between the different groups (*p <* 0.05), they were all within the normal range, as shown in Table 1.

### Comparison of alleles and acetylation types

The analysis of alleles and acetylation types in the study participants indicated that their distribution types were statistically different between groups (*p* < 0.05), and in patients with grade 4 liver injury, either two alleles of *6 or *7 were present. Liver injuries occurred in 42.4% (14/33) of patients with fast acetylation genotypes, 55.2% (32/58) of those with intermediate acetylation genotypes and 65.5% (19/29) of patients with slow acetylation genotypes. All patients with fast acetylation genotypes had mild liver injuries, and the proportion of patients with slow acetylation genotypes who had grade 4 liver injury was higher than those with intermediate and fast acetylation genotypes.

These results confirmed that patients with slow acetylation genotypes containing any two alleles of *6 and *7 may have a higher rate of liver failure than patients with other allele types. None of the patients exhibited grade 3 or grade 5 liver injury. For additional details, see Table 2.

**TABLE 2:** Comparison of alleles and acetylation types in groups with different degrees of liver injury and none (grade 0).

Variables	Total (n = 120)	0 (n = 55)	1 (n = 55)	2 (n = 7)	4 (n = 3)	Statistics	*P*
allel						Fisher	0.024
*4*4	33 (27.5)	19 (34.5)	14 (25.5)	0 (0)	0 (0)		
*5*4	8 (6.7)	5 (9.1)	3 (5.5)	0 (0)	0 (0)		
*6*4	27 (22.5)	12 (21.8)	13 (23.6)	2 (28.6)	0 (0)		
*6*5	3 (2.5)	0 (0)	3 (5.5)	0 (0)	0 (0)		
*6*6	8 (6.7)	3 (5.5)	2 (3.6)	2 (28.6)	1 (33.3)		
*6*7	13 (10.8)	6 (10.9)	6 (10.9)	0 (0)	1 (33.3)		
*7*4	23 (19.2)	9 (16.4)	13 (23.6)	1 (14.3)	0 (0)		
*7*7	5 (4.2)	1 (1.8)	1 (1.8)	2 (28.6)	1 (33.3)		
Types of acetylation						Fisher	0.022
Fast acetylation	33 (27.5)	19 (34.5)	14 (25.5)	0 (0)	0 (0)		
slow acetylation	29 (24.2)	10 (18.2)	12 (21.8)	4 (57.1)	3 (100)		
Intermediate acetylation	58 (48.3)	26 (47.3)	29 (52.7)	3 (42.9)	0 (0)		

### Comparison of the time before liver damage and the recovery of liver function in patients with different acetylation types

The results showed that the time to observing liver damage and the time to liver function recovery did not differ statistically between the different groups (*p* > 0.05). The time before liver damage occurred earlier than the overall mean time in patients with slow acetylation genotypes, and the time required for the recovery of liver function was shorter than the overall mean time in patients with fast acetylation genotypes, as shown in Table 3.

**TABLE 3:** Comparison of time before liver damage and liver function recovery time in patients with different acetylation types.

Variables	Total (n = 120)	Fast acetylation (n = 33)	Slow acetylation (n = 29)	Intermediate acetylation (n = 58)	Z	*P*
Time before liver damage (day)	11 (6, 24)	12 (6, 27)	7 (6, 18)	19.5 (5.25, 27.25)	1.044	0.593
Time of liver function recovery (day)	12 (7, 18)	7 (7, 12)	14 (7, 31.5)	14 (7, 17.5)	3.283	0.194

## Discussion

In countries with a high rate of TB treatment, ATDILI is a major problem. Existing studies have focused on patients with genetic polymorphisms and altered genes encoding metabolic enzymes for INH bioactivation and inactivation, leading to the differential accumulation of INH-active metabolites and resulting in liver injury (Zhou et al., 2022; Bagcchi, 2023). Currently, with the discovery of new mechanisms, the relevance of INH-mediated mitochondrial dysfunction in ATDILI is gradually being recognised, but the exact mechanism of its occurrence remains unclear (Jiang et al., 2021). This study investigated the correlation between NAT2 genetic polymorphisms and ATDILI in Chinese patients with TB.

Personalised dosing therapy based on drug-metabolising enzymes and transporter genomes has become one of the focuses of personalised medicine. To study the association between NAT2 genetic polymorphisms and ATDILI, many countries throughout the world have conducted a series of studies. Yuliwulandari Rika et al. (Yuliwulandari et al., 2019) performed NAT2 genotyping by direct DNA sequencing in 100 cases of clinically severe ATDILI and 210 non-ATDILI controls; they found that slow NAT2 acetylation was significantly associated with ATDILI risk, while fast and intermediate acetylation was associated with reduced ATDILI risk, thus suggesting the importance of NAT2 genotype and phenotype determination for reducing ATDILI risk. Chamorro et al. (Chamorro et al., 2012) found that slow acetylation increased the risk of ATDILI in their study of 185 patients with TB in Argentina. Li Xinjie et al. (Li et al., 2011) and Shen TT et al. (Shen et al., 2015) found that the genetic phenotype of NAT2 in the Chinese Han population of patients with TB was predominantly intermediate, and the risk of drug-related liver injury was higher in the slow metabolic form of NAT2. The participants in this study were all Han Chinese, and the genetic phenotype of NAT2 was found to be predominantly intermediate (48.3%), which was consistent with the results of Xinjie Li et al.‘s research (Li et al., 2011)*.* A study conducted by Toure et al. (Toure et al., 2016) confirmed that NAT2 fast acetylator genotypes accounted for a high proportion in Senegalese patients with TB. The current results showed that the NAT2 fast acetylation genotype was the least predominant. Overall, these studies may reflect that polymorphism of the *NAT2* gene is related to racial regional differences. Therefore, we need to study the association between NAT2 genetic polymorphisms and ATDILI to provide a theoretical basis for personalised drug delivery in mainland China.

Yang Seungwon et al. (Yang et al., 2019) reported that ATDILI was more likely to occur in patients with NAT2 slow acetylation genotypes, who may require close monitoring. In addition, this study found that patients with the slow acetylation genotypes had a higher rate of liver injury than patients with intermediate and fast acetylation genotypes; patients with slow acetylation genotypes developed liver damage earlier than the overall mean time, and all three cases of liver failure occurred in patients with slow acetylation genotypes. Accordingly, slow acetylation types have a greater risk of causing ATDILI.

Suvichapanich Supharat et al. (Suvichapanich et al., 2018) conducted a meta-analysis of 18 studies involving 822 cases of ATDILI and 4,630 controls; they confirmed a strong association between each NAT2 slow acetylation genotype and ATDILI, except for NAT2*5B/*5B. Furthermore, a meta-analysis also argued that a personalised clinical drug dosage model is especially important for the populations of South and East Asia with a high incidence of ATDILI.

Additional *in vitro* studies with INH as a substrate provided support for the presence of ultralow acetylation alleles (NAT2*6A and NAT2*7B). In Thailand, a study concluded that NAT2 slow acetylation genotypes are a high risk factor for drug-induced liver injury in patients with TB (Wattanapokayakit et al., 2016). In Japan, Higuchi et al. (Higuchi et al., 2007) found that slow acetylation genotype NAT2*6 could increase hepatotoxicity in patients with TB, while acetylation genotype NAT2*4/*4 could reduce the risk of liver injury in such patients. The results of this study found that patients with grade 4 liver injury (liver failure) had either *6 or *7 alleles, and patients with grade 2 liver injury had either *6 or *7 alleles; it was hypothesised that patients with slow acetylation genotypes containing either *6 or *7 alleles may have a higher rate of liver failure than those with other types of alleles.

The above studies reflect the difference in dominant sub-genotypes of *NAT2* between countries and races, and further investigation is required in relevant studies.

The correlation between genetic polymorphism and changes in serum enzyme expression levels depends on several factors, including genotype, environmental factors and genetic interactions. In molecular biology, genetic polymorphism refers to the presence of different alleles of the same gene in a population, which may affect gene expression and function and lead to biological differences between individuals. These genotype differences may lead to changes in enzyme expression levels, affecting the metabolism and other biological processes. Some genetic polymorphisms have been shown to be correlated with the expression levels and activity of specific enzymes. For example, single-nucleotide polymorphisms have been found to be correlated with the expression levels of some metabolic enzymes in certain genes. In addition, other types of genetic variations, such as insertions/deletions or gene locus amplification, may also affect enzyme expression and function.

However, the correlation between genetic polymorphism and enzyme expression levels is also influenced by environmental factors and genetic interactions. For example, diet, drug exposure and environmental factors may affect enzyme expression levels, altering the relationship between genotype and enzyme expression (Scott, 2010). Additionally, genetic interactions may also affect the relationship between genetic polymorphism and enzyme expression levels. Therefore, more research is needed to determine the relationship between genetic polymorphism and enzyme expression levels; furthermore, environmental factors need to be controlled and genetic interactions considered.

In recent years, due to an increase in the combination of drugs, drug-related liver injury has gradually become a common clinical pharmacogenetic disease, with a serious impact on treatment effects and the quality of life of patients (Liu et al., 2019). The long treatment period and many adverse reactions that may arise during the treatment of patients with TB often lead to treatment interruptions or regimen changes, resulting in reduced efficacy and drug resistance and directly impacting the effectiveness of TB control.

There are few studies on NAT2 genetic polymorphisms and a lack of research on the correlation between NAT2 genetic polymorphisms and ATDILI in China. It has been reported that a personalised clinical drug dosage model is especially important for the populations of South and East Asia with a high incidence of ATDILI; thereby, we investigated the association between NAT2 genetic polymorphisms and ATDILI in China. The present study has important implications for identifying patients with a high risk of developing liver damage before anti-TB treatment and has clinical implications for the targeted guidance of individualised drug therapy, the mitigation of ATDILI and the rational distribution and use of drugs, which will benefit patients with ATDILI in China.

Herein, we found that in 33 patients with adaptive liver injury (grade 1 liver injury could be recovered by continuing HRZE treatment), there were 12 cases (36.4%) of fast acetylation genotypes, 16 cases (48.5%) of intermediate acetylation genotypes and five cases (15.1%) of slow acetylation genotypes. In 32 patients with a modified anti-TB treatment plan were two cases of fast acetylation genotypes (6.25%), 16 cases of intermediate acetylation genotypes (50%) and 14 cases of slow acetylation genotypes (43.75%). Therefore, we speculated that the adaptability of fast acetylation genotypes was stronger than that of slow and intermediate acetylation genotypes; consequently, patients with fast acetylation genotypes could continue to be treated with the HRZE regimen under the condition of monitoring liver function and coagulation function after the emergence of grade 1 liver injury.

Previous studies have found that polymorphisms in the *NAT2* gene are most likely to be associated with the anti-TB drug INH (Huang et al., 2021). One study described the liver damage mechanism of INH, RFP and PZA (Tuberculosis Branch of Chinese Medical Association, 2019). In this study, among 30 patients (intermediate and slow acetylation genotypes) with a modified anti-TB treatment regimen, 20 patients were re-used INH, among which, 15 patients were treated with rifapentine (0.6 twice a week), and only four patients were re-used PZA. Twenty-five patients were treated with moxifloxacin (0.4 once a day) or levofloxacin (0.5 once a day). No liver injury was found at follow-up.

It is speculated that the combination of INH with RFP and PZA results in a superposition effect (especially in slow acetylation), and INH combined with rifapentine, moxifloxacin or levofloxacin in the treatment of TB may reduce the probability of liver injury. Rifapentine is a new long-acting rifamycin antibiotic, with a good antibacterial effect on *Mycobacterium* TB. Compared with the widely used first-line anti-TB drug RFP, its antibacterial spectrum is similar, but its anti-TB action is 2–10 times higher, with fewer adverse reactions, a longer elimination half-life in plasma and a slightly smaller induction effect of cytochrome P450. However, its hepatotoxicity is still substantial, especially in TB with liver injury, and there are more obvious individual differences.

This study has some limitations. First, the sample size was relatively small, and second, no pharmacokinetic data were included. Furthermore, the association between the expression level of NAT2 and the occurrence of ATDILI still requires further investigation.

## Conclusion

This study found that NAT2 genetic polymorphisms were associated with the development of ATDILI in Chinese patients with TB, and patients with slow acetylation genotypes had higher rates of liver injury and failure than those with intermediate and fast acetylation genotypes. Additionally, patients with slow acetylation genotypes containing any two alleles of *6 and *7 had higher rates of liver failure than those with other alleles. These results are in line with previous findings. However, the sample size of the liver failure group in this study was small, which had a certain influence on the conclusion. In subsequent studies, we will expand the sample size to further verify the conclusions of this paper.

This study has important implications for identifying patients with a high risk of developing liver damage before anti-TB treatment and has important clinical implications for the targeted guidance of individualised drug therapy, which will benefit patients with ATDILI in China. If the association between genetic polymorphisms and the risk of ATDILI is determined, a personalised clinical drug dosage model could be developed for the treatment of TB.

## Data Availability

The original contributions presented in the study are included in the article/Supplementary Material, further inquiries can be directed to the corresponding author.
