# Supplementary material for: N-acetyltransferase 2 genetic polymorphisms and anti-tuberculosis-drug-induced liver injury: a correlation study
Source: Front Pharmacol. 2023 Aug 31;14:1171353. doi: 10.3389/fphar.2023.1171353 (PMC10501134; doi:10.3389/fphar.2023.1171353)
Supplement: Supplementary file 1 [file Table1.DOCX]

Table 1 Comparison of clinical baseline data between the groups with different degrees of liver impairment and the control group (groups without liver impairment)

| Variables | Total (n = 120) | 0 (n = 55) | 1 (n = 55) | 2 (n = 7) | 4 (n = 3) | F/Z/χ^2^ | *P* |
| --- | --- | --- | --- | --- | --- | --- | --- |
| sex |  |  |  |  |  | Fisher | 0.379 |
| man | 88 (73.3) | 42 (76.4) | 39 (70.9) | 6 (85.7) | 1 (33.3) |  |  |
| woman | 32 (26.7) | 13 (23.6) | 16 (29.1) | 1 (14.3) | 2 (66.7) |  |  |
| age | 55.5 (36, 66) | 56 (41.5, 65.5) | 53 (36, 67) | 58 (53.5, 70.5) | 25 (21, 45.5) | 2.366 | 0.500 |
| diabetes mellitus |  |  |  |  |  | Fisher | 0.615 |
| deny | 95 (79.2) | 43 (78.2) | 42 (76.4) | 7 (100) | 3 (100) |  |  |
| yes | 25 (20.8) | 12 (21.8) | 13 (23.6) | 0 (0) | 0 (0) |  |  |
| hypertension |  |  |  |  |  | Fisher | 0.782 |
| deny | 95 (79.2) | 45 (81.8) | 42 (76.4) | 6 (85.7) | 2 (66.7) |  |  |
| yes | 25 (20.8) | 10 (18.2) | 13 (23.6) | 1 (14.3) | 1 (33.3) |  |  |
| ALT (U/L) | 14 (9, 22) | 11 (7.5, 15.5) | 17 (11, 27) | 14 (9, 32) | 8 (6.5, 17.5) | 12.727 | 0.005 |
| AST (U/L) | 19 (14.5, 26) | 16 (13.5, 22) | 22 (17, 27.75) | 26 (16.5, 26.5) | 20 (17, 29.5) | 11.084 | 0.011 |
| Tbil (μmol/L) | 10.4 (7.3, 14.95) | 9.9 (6.25, 13.7) | 10.35 (7.45, 14.12) | 13.1 (11.75, 22.7) | 15.1 (14.15, 16.55) | 8.294 | 0.040 |
| INR | 1.04 (0.97, 1.07) | 1.04 (0.98, 1.08) | 1.03 (0.97, 1.06) | 1.02 (0.96, 1.11) | 1.04 (1.04, 1.06) | 0.810 | 0.847 |
| PTA (%) | 104.2 ± 12.5 | 102.9 ± 11.8 | 104.7 ± 12.2 | 106 ± 22.2 | 106.7 ± 15.3 | 0.199 | 0.896 |

Note: ALT, alanine aminotransferase; AST, glutamic oxalacetic transaminase; TBil, total bilirubin; INR, international normalised ratio; PTA, prothrombin time activity. Normal range: ALT (7-40 U/L); AST (13-35 U/L); Tbil (0-21 μmol/L); INR (0.8-1.1); PTA (60-160 %).

Table 2 Comparison of alleles and acetylation types in groups with different degrees of liver injury and none (grade 0)

| Variables | Total (n = 120) | 0 (n = 55) | 1 (n = 55) | 2 (n = 7) | 4 (n = 3) | statistics | *P* |
| --- | --- | --- | --- | --- | --- | --- | --- |
| allel |  |  |  |  |  | Fisher | 0.024 |
| *4*4 | 33 (27.5) | 19 (34.5) | 14 (25.5) | 0 (0) | 0 (0) |  |  |
| *5*4 | 8 (6.7) | 5 (9.1) | 3 (5.5) | 0 (0) | 0 (0) |  |  |
| *6*4 | 27 (22.5) | 12 (21.8) | 13 (23.6) | 2 (28.6) | 0 (0) |  |  |
| *6*5 | 3 (2.5) | 0 (0) | 3 (5.5) | 0 (0) | 0 (0) |  |  |
| *6*6 | 8 (6.7) | 3 (5.5) | 2 (3.6) | 2 (28.6) | 1 (33.3) |  |  |
| *6*7 | 13 (10.8) | 6 (10.9) | 6 (10.9) | 0 (0) | 1 (33.3) |  |  |
| *7*4 | 23 (19.2) | 9 (16.4) | 13 (23.6) | 1 (14.3) | 0 (0) |  |  |
| *7*7 | 5 (4.2) | 1 (1.8) | 1 (1.8) | 2 (28.6) | 1 (33.3) |  |  |
| Types of acetylation |  |  |  |  |  | Fisher | 0.022 |
| Fast acetylation | 33 (27.5) | 19 (34.5) | 14 (25.5) | 0 (0) | 0 (0) |  |  |
| slow acetylation | 29 (24.2) | 10 (18.2) | 12 (21.8) | 4 (57.1) | 3 (100) |  |  |
| Intermediate acetylation | 58 (48.3) | 26 (47.3) | 29 (52.7) | 3 (42.9) | 0 (0) |  |  |

Table 3 Comparison of time before liver damage and liver function recovery time in patients with different acetylation types

| Variables | Total (n = 120) | Fast acetylation (n = 33) | Slow acetylation (n = 29) | Intermediate acetylation (n = 58) | Z | *P* |
| --- | --- | --- | --- | --- | --- | --- |
| Time before liver damage (day) | 11 (6, 24) | 12 (6, 27) | 7 (6, 18) | 19.5 (5.25, 27.25) | 1.044 | 0.593 |
| Time of liver function recovery (day) | 12 (7, 18) | 7 (7, 12) | 14 (7, 31.5) | 14 (7, 17.5) | 3.283 | 0.194 |

Supplementary Table 1 Primers for PCR

| Name |  | Primers for PCR (5’-3’) |
| --- | --- | --- |
| NAT2-F-282 | PS02001PF | CCACAATCGGTTTTCAGACCA |
|  |  |  |
| NAT2-R-282 | PS02001PR | AAGGTGAACCATGCCAGTGCT |
|  |  |  |
| NAT2-S-282 | PS02001PS | CAATGTTAGGAGGGTATTT |
|  |  |  |
| NAT2-F-341 | PS02002PF | CAAATACAGCACTGGCATGGTTC |
|  |  |  |
| NAT2-R-341 | PS02002PR | GAAAATGCAAGGCACCTGAG |
|  |  |  |
| NAT2-S-341 | PS02002PS | CTCCTGCAGGTGACCA |
|  |  |  |
| NAT2-590-F-5 | PS02003PF | GACCAAATCAGGAGAGAGCAGTAT |
|  |  |  |
| NAT2-590-R-5 | PS02003PR | CCACCAAACAGTAAACCCCTTCT |
|  |  |  |
| NAT2-590-S-5 | PS02003PS | TAGACTCAAAATCTTCAATT |
|  |  |  |
| NAT2-F-857 | PS02004PF | GTGGGCTTCATCCTCACCTATAGA |
|  |  |  |
| NAT2-R-857 | PS02004PR | CGTGAGGGTAGAGAGGATATCTGA |
|  |  |  |
| NAT2-S-857 | PS02004PS | CCAAACCTGGTGATG |
|  |  |  |
